# Extended Spectrum *β*-Lactamases among Gram-Negative Bacterial Isolates from Clinical Specimens in Three Major Hospitals in Northern Jordan

**DOI:** 10.1155/2009/513874

**Published:** 2009-09-10

**Authors:** Raymond G. Batchoun, Samer F. Swedan, Abdullah M. Shurman

**Affiliations:** Department of Medical laboratory Sciences, Faculty of Applied Medical Sciences, Jordan University of Science and Technology, P.O. Box 3030 Irbid 22110, Jordan

## Abstract

*Background and Objectives*. Extended spectrum *β*-lactamase (ESBL) production is increasing all over the world, and organisms other than *E. coli* and *K. pneumoniae* are acquiring this character. ESBL production is detectable by automation, E-test, double disk diffusion (DDD), and PCR. This study aimed to determine the prevalence of ESBL production among clinical isolates of gram-negative rods, and to evaluate the effectiveness of augmentation of clavunate with Cefotaxime, Ceftazoxime, Aztreonam, Ceftriaxone, and Cefpodoxime in detecting ESBL production. *Methods*. 472 clinical gram-negative isolates identified by standard methods were tested for ESBL-production by (DDD) method using six cephalosporins and amoxicillin-clavulinate discs. *Results*. 108/472 (22.9%) of the isolates were ESBL producers, and were prevalent in tertiary care hospitals. 88.2% of *E. cloacae*, 71.4% of *K. pneumoniae*, 28.6% of *K. oxytoca*, 12.5% of *C. freundii*, 11.1% of *A. calcoacceticus*, and 10.8% of *E. coli* were ESBL producers. The DDD test demonstrated some variations in the efficacy of the different cephalosporins in detecting all the ESBL producers. The inclusion of ceftizoxime discs increased the efficacy of the test. It is concluded that ESBL-producing bacteria were prevalent among our hospitalized patients, and involved genera other than *Klebsiella* and *Escherichia*, and the inclusion of ceftizoxime increased the efficacy of ESBL detection by the DDD test.

## 1. Introduction

 Among the family Enterobacteriacae, the production of plasmid-mediated extended-spectrum *β*-lactamase (ESBLs) has emerged as an important mechanism of resistance to *β*-lactam drugs that account for approximately 50% of antibiotic consumption [1]. The vast majority of ESBLs are derivatives of TEM-1 and TEM-2 chromosomally encoded enzyme of *Klebsiella pneumoniae* [2]. These enzymes are capable of hydrolyzing a wide range of *β*-lactams, including most recently developed cephalosporins, but are not active against cephamycins and carapenems [3]. In interpreting the phenotype of ESBL-positive strains with regard to *β*-lactams, it should be borne in mind that drug resistance may also result from the combined activity of a specific ESBL together with other *β*-lactamases (the chromosomal AmpC) [3], or the plasmid-borne one [4].

Among Enterobacteriacae, ESBLs have been found mainly in *Klebsiella* spp. and *Escherichia coli*, but have been also reported in other genera world wide, such as *Citrobacter, Enterobacter, Morganella, Proteus, Providencia, Salmonella, Serrati,* and *Pseudomonas* [5–9]. 

Infections caused by ESBL-producing bacteria often involve immune-compromised patients, making it difficult to eradicate these organisms in high-risk wards, such as intensive care unites [10, 11].

Microbiology laboratories play an important role in detecting and promptly reporting the isolation of ESBL-positive bacteria, especially AmpC beta-lactamases-producing ones that complicate therapy and limit treatment options [12]. Drug susceptibility data are of major importance for the clinical management of patients infected by these organisms [13]. Reduced susceptibility or increase in resistance to extended-spectrum cephalosporins and/or monobactams represents the first indicator of ESBL production, but confirmation is dependent on synergy between clavulanate and the selected *β*-lactams, using double-disk diffusion method, or E-test [14]. The expression of an extended-spectrum enzyme does not always involve a phenotype that can be interpreted as resistant by the routine MICs and disk diffusion methods that follow current National Committee for Clinical Laboratory Standards/recently named Clinical and Laboratory Standards Institute (NCCLS/CLSI) breakpoint. Accordingly, ESBL-positive strains should be reported as resistant even if drug MICs are below breakpoints established for cephalosporins and aztreonam. This is defined for both *Klebsiella* spp, and *E.coli*, but not established for the other Enterobacteriaceae [15].

When detecting ESBL-positive strains, microbiology laboratories should provide the clinician with reliable therapeutic options for successfully treating infected patients, since ESBL-distribution has been shown to differ among countries [3, 6, 16]. Thus monitoring of the prevalence and the types of extended spectrum *β*-Lactamase enzymes may contribute to defining the degree of the problem in a specific geographical area, and establishing a proper treatment protocol.

Before the emergence and increased spread of ESBL-producing gram-negative bacteria, most infections could be reliably treated with second- and third-generation cephalosporins. However, ESBL-producing organisms are spreading world wide, resulting in the failure of empiric therapy dependent on second- and third-generation cephalosporins which complicate infections in immune-compromised patients, neonates, the elderly, debilitated patients, nosocomial infections, and outbreaks occurring in hospital setting [17–22]. 

The aim of this study was to assess the prevalence of ESBL-producing gram-negative organisms among different bacterial genera and species isolated from clinical cases from three major hospitals in northern Jordan, and to assess the effectiveness of clavulanate and six cephalosporins in detecting ESBL production.

## 2. Materials and Methods

### 2.1. Bacterial Isolates

A number of 472 gram-negative bacterial isolates recovered from 463 patients were included in this study. These isolates were isolated from clinical specimens at the microbiology laboratories in three hospitals in northern Jordan during ten-month period (January–October 2004). These hospitals were King Abdullah University Teaching Hospital (KAUTH) (tertiary care hospital), Princess Basma Teaching Hospital (PBTH) (primary and secondary care hospital), and Princess Rahmeh Teaching Hospital (PRTH) (secondary care hospital dealing with pediatrics, neonates, obstetrics and gynecology, and immune-suppressed patients). Gram-negative bacteria belonging to the genera *Escherichia, Klebsiella, Proteus, Citrobacter, Enterobacter, Serratia, Pseudomonas, Acinetobacter, Alkaligenes,* and *Stenotrophemonas* were included in the study. These bacteria were isolated from various clinical specimens such as blood culture, urine, CSF, sputum, wound, pus, ear swabs, eye swabs, peritoneal fluid, and other miscellaneous sources. These isolates were recovered from the specimens by being cultured on the specific media, under the optimal conditions, and were identified to genus and species using standard methods (conventional manual methods, Rapid-ID—Remel, USA, and Vitek automation technology) [23–25]. Multiple samples or isolates from the same patient were excluded from the study. These isolated were stored on simple storage media as described by Evans et al. in 1977 [26].

### 2.2. ESBL Production

Test organisms from stock culture were activated by inoculation in to Mueller Hinton broth (BD, USA) and incubated at 37°C for 24 hours. The concentration of the bacterial suspension was adjusted to be equivalent to 0.5 McFarland standards. The test organism was seeded on the surface of freshly prepared Mueller Hinton agar (BD, USA), in three directions using a sterile Dacron swab, according to the recommendations of Kirby-Bauer Disk Diffusion method [25], and NCCLS (CLSI) guide lines [27]. The plates were allowed to stand at room temperature for 15 minutes prior to the application of antibiotic containing disks. 

 ESBL-producing isolates were detected using the Double Disk Diffusion method (Double Disk Approximation Test) [28]. Six antibiotic disks (Cefotaxime 30 ug, Ceftazidime 30 ug, Ceftriaxone 30 ug, Cefpodoxime 10 ug, Ceftizoxime 30 ug, and Aztreonam 30 ug) (Oxoid, UK) were placed around a central disk of Amoxicillin-Clavulanic acid 30 ug (20:10 resp.) (Oxoid, UK), 30 mm center to center on Mueller Hinton agar plates seeded with organism being tested for ESBL production.

Plates were incubated aerobically at 37°C for 18–24 hours, and the diameter of the inhibition zone (if any) around the antimicrobial disks was measured in mm using a ruler. Any augmentation (increase in diameter of inhibition zone) between the central Amoxicillin-Clavulanic acid disk and any of the six antibiotic disks showing resistance or intermediate susceptibility was recorded, and the organism was thus considered as an ESBL producer.

## 3. Results

### 3.1. Distribution of Bacterial Species with Sample Source

Urine was the major source of the bacterial isolates collected, comprising 262/472 (56%) of the total isolates, blood culture 79/472 (16.7%), swabs from various sites 80/472 (16.9%), CSF 16/472 (3.3%), sputum 8/472 (1.6%), and the other miscellaneous sources 44/472 (5.5%) (Table 1).

*Escherichia coli* was the most common species isolated from these specimens, comprising 195/472 (41.4%). This organism was the major isolate recovered from urine samples, representing 171/195 (87.7%) of the total *E. coli* isolates from the three teaching hospitals (Table 1). 

Genus *Klebsiella* was the second isolated pathogen from the tested samples, constituting 132/472 (28%) of the total isolates, of which *Klebsiella pneumonia* represented 84/472 (17.8%) of the total isolates. However, 48/84 (57%) of the *Klebsiella pneumoniae* isolates were recovered from blood culture, 21/84 (25%) from urine, and the remaining 15/84 (18%) from sputum and the other miscellaneous sources. Similarly, *Klebsiella oxytoca* comprised 28/472 (5.9%) of the total isolates, of which 19/28 (67.9%) were isolated from urine, and the remaining 9/28 (32.1%) were detected at low frequency from the specimens (Table 1).

*Pseudomonas aeruginosa* represented 64/472 (13.5%) of the total isolates, where 24/64 (37.5%) of these *pseudomonas aeruginosa *were isolated from swabs of various sources, 12/64 (18.8%) from Cerebrospinal fluid (CSF), 10/64 (15.6%) from urine, and the remaining 18/64 (28.1%) were from the other miscellaneous sources (Table 1). Additionally, 16/472 (3.4%) *pseudomonas spp* other than *Pseudomonas aeruginosa* were isolated mainly from various swabs, urine, and the other miscellaneous samples.

*Proteus mirabilis* isolates represented 23/472 (4.8%) of the bacterial isolates, where 16/23 (69.5%) of them were isolated from urine samples, 7/23 (30.4%) from swabs of various sources and other miscellaneous specimens (Table 1).

Genus *Enterobacter* was common among the gram-negative isolates constituting 32/472 (7.8%) of the isolates. *Enterobacter cloacae* was the major species isolated constituting 11/17 (64.7%) of the *Enterobacter* recovered from blood cultures, 5/17 (29.7%) from urine cultures, and 1/17 (5.9%) from sputum samples. The other 14 *Enterobacter spp* were isolated from swabs of various sources and urine (Table 1).

Bacterial isolates belonging to the genus *Acinetobacter* (*Acinetobacter baumannii*, *Acinetobacter calcoaceticus*, *Acinetobacter lwoffii,* and other *Acinetobacter spp*) constituted 18/472 (3.8%) most of which were recovered from blood culture, sputum, and miscellaneous sources. Other gram-negative bacterial species, including *Citrobacter frundii*, *Serratia marcescens*, *Alkaligenes xylosoxidans*, and *Stenotrophomonas maltophilia,* collectively represented 12/472 (2.5%), most of which were isolated from urine samples (Table 1). 

### 3.2. ESBL Production

 The Double Disk diffusion test revealed that out of the 472 gram-negative isolates included in the study, 108 isolates were ESBL producers. These ESBL-producing bacteria belonged to 8 different species out of the 18 species isolated from the clinical samples from the three teaching hospitals (Table 2).

ESBL production was very common among *Klebsiella pneumoniae*, where out of the 84 *Klebsiella pneumoniae *isolates, 49 (71.4%) were ESBL producers. However, within *Klebsiella oxytoca *isolates, only 8 out of 28 isolates were ESBL-producers, representing only 28.5%. On the other hand, ESBL production in *E. coli *was found in 21/195 isolates representing only 10.8% (Table 2).

Out of the 17 *Enterobacter cloacae* isolated, 15 (88.2%) were ESBL producers, and all of them were recovered from blood culture and urine culture from KAUTH during *Enterobacter cloacae* outbreak (Table 2). 

ESBL production in *Citrobacter frundii* was found only in one isolate out of the 8 isolates (12.5%) recovered from KAUTH. Similarly, only one *Acinetobacter calcoaceticus* out of the nine isolates was ESBL producer. None of the other *Acinetobacter spp*. isolated produced ESBL (Table 2). Further more, one isolate of both Enterobacter *hormaechei* and *Stenotrophemonas maltophilia* was ESBL producer (Table 2). Most of the ESBL-producing isolates were recovered from patients samples collected from the tertiary care KAUTH.

### 3.3. Effectiveness of Antibiotics Used in Detecting ESBL Production

The most important feature in detecting ESBL-producing isolates by the Double Disk Diffusion method is the formation of augmentation of the bacterial growth inhibition zone between the central Amoxicillin-Clavulanic acid (AMC) disk and the surrounding cephalosporin's ones. The 21 ESBL-producing *Escherichia coli* isolates gave the highest number of augmentation zones when Cefotaxime (CTX) and Ceftizoxime (ZOX) disks were used. Both of these antibiotics were successful in identifying 20/21 (95.2%) of the ESBL producers. Augmentation with Aztreonam (ATM) detected 18/21 (85.7%) ESBL-producing *E. coli*, Ceftazidime (CAZ) 15/21 (71.4%), Ceftriaxone (CRO) 11/21 (52.4%), and Cefpodoxime (CPD) 4/21 (19%) (Table 3).

 ESBL-producing *Enterobacter cloacae*, which were collected during an out break in KAUTH, had little variation in the antibiogram of the isolates suggesting a common source. Disks used in the detection of ESBL were closely successful in detecting ESBL production, where ATM was capable of detecting all of the 15 ESBL producing isolates, followed by CTX and CRO which detected 14/15 (93.3%) of them. Both CAZ and ZOX detected only 13/15 (86.7%), while CPD detected only 12/15 (80%) of the ESBL-producing *Enterobacter cloacae* (Table 3).

*Klebsiella pneumoniae* isolates producing ESBL gave augmentation with ZOX, where it detected 57/60 (95%) of the ESBL-producing isolates, CTX 49/60 (81.7%), ATM 43/60 (71.7%), CAZ 30/60 (60%), CRO 26/60 (43.3%), and CPD 15/60 (16.7%), respectively, (Table 3). Similarly, out of the 8 ESBL-producing *Klebsiella oxytoca*, ZOX detected 7/8 (87.5%) of the ESBL producers, ATM 6/8 (75%), CTX 4/8 (50%), CAZ 3/8 (37.5%), and CRO 2/8 (25%), respectively, (Table 3).

On the contrary, effectiveness of the antibiotics used in the detection of ESBL production in *Acinetobacter calcoaceticus, Citrobacter frundii, Enterobacter hormaechei,* and *Stenotrophemonas maltophilia* could not be established due to the small number of ESBL-producing isolates among these species (Table 3).

## 4. Discussion

This study included three major hospitals in northern Jordan, to assess the prevalence and distribution of ESBL-positive species among *Enterobacteriaceae* and other gram-negative bacteria recovered from clinical specimens, and evaluated the efficacy of different cephalosporins in the detection of ESBL production. 

 In a study between 1990 and 1993, Youssef et al., in 1999 reported that 38% of *Klebsiella pneumoniae* isolates were ESBL producers [29]. Shehabi et al., in 1999 reported an incidence of ESBL production in *Klebsiella pneumoniae* and *Escherichia coli* isolates in the ICU of Jordan University Hospital, to be 70% and 38%, respectively, [30]. Batchoun and Matalka (unpublished data) did not detect any ESBL producers among various pseudomonas species. 

Extended-spectrum *β*-lactamases (ESBLs) are enzymes capable of hydrolyzing oxyimino-cephalosporins, such as Cefotaxime (CTX), Ceftriaxone, Ceftazidime (CAZ), and monobactams (e.g., aztreonam (ATM)), thereby causing resistance to these drugs. The enzymes are detected most commonly in *Klebsiella pneumoniae* and *Escherichia coli* but have been noted in other members of the family *Enterobacteriaceae* as well [22]. The majority of these enzymes usually have only 1, 2, or 3 amino acid mutation from those of the parent enzymes (TEM-1, TEM-2, and SHV-1). These mutations are thought to have evolved under selective pressures exerted by antibiotic treatment, and continued use of cephalosporin antibiotics. The fact that most of these enzymes are carried by plasmids has facilitated the spread of ESBL enzymes among members of the family *Enterobacteriaceae* and other gram-negative bacteria, and single strain of *K. pneumoniae or E. coli* may harbor different variants of ESBL genes such as SHV with CTX-M type, AmpC or metalo-*β*-lactamases together, that may complicate therapy [31]. 

In our study, urine was the source of 262/472 (56%) of the isolates, indicating that UTI is a common illness in our community. Blood culture (16.7%, 79/472) was the second major source of isolates thus indicating the relatively high frequency of gram-negative bacteria involved in bacteremias or septicemias in hospitalized patients. The rest of the isolates collected in our study were from ear swabs, swabs from miscellaneous sources, CSF, and sputum.

Out of the 472 isolates recovered in this study, 195 isolates were *E. coli* and 84 were *K. pneumoniae*. Both *E. coli* and *K. pneumoniae* are involved world wide in ESBL production, however, as major isolates from our hospitals, they can give us a good picture about ESBL production in our community.

Other clinically important isolates that include *Pseudomonas aeruginosa* (64/472), *Klebsiella oxytoca* (28/472), *Proteus mirabilis* (23/472), *Enterobacter cloacae* (17/472), and *Pseudomonas Spp* (16/472) were recovered in moderate number, whereas the remaining isolates were scarce. Although the moderate number of these isolates is high enough to allow a calculation of the percentage of ESBL production, this percentage may not actually reflect the true nature of ESBL production in the isolate nation wide, and may only indicate the presence of an endemic bacteria in a specific hospital or a specific ward. This is certainly true—if not at least—for the 17 isolates of *Enterobacter cloacae* which were all collected from King Abdullah University Teaching Hospital during an outbreak. 

In regard to ESBL production, one of the most alarming findings of this study is the ESBL production in 60/84 (72.4%) of the *Klebsiella pneumoniae* isolates. This percentage is considered to be very high compared to prevalence of ESBL production world wide among this species when compared with the 20% prevalence of the Italian study [32], and 39.5% in the Chinese study [33], 11.3% in Saudi Arabian study [34], and 13.3% in the Kuwaiti study [35]. However, our results are matching previous Jordanian study which reported that 70% of the *K. pneumoniae* isolates recovered from the ICU of Jordan University Hospital to be ESBL producers [30]. On the other hand, none of the *Klebsiella pneumoniae* isolates recovered from Princess Basma Teaching Hospital were ESBL producer, which may be attributed to the low number of isolates recovered, the nature of the hospital type, and the patients being served. On the contrary, Isolates from Princess Rahma Teaching Hospital and King Abdullah University Teaching Hospital showed ESBL production in 35/39 (89.7%) and 25/32 (78.1%), respectively. The abnormally high percentage of ESBL production in these two hospitals may indicate the presence of a previously undetected source of a nosocomial infection, the nature of patients being served as both hospitals are serving debilitated patients, and as referral hospitals for malignancy and chronic diseases. Such findings impose the need for applying specific infection control measures to eliminate this organism.

One of the highest ESBL producers by percentage was found to be *Enterobacter cloacae*, which was found in 15/17 collected isolates, thus constituting 88.2%. As indicated before, this high percentage disagrees with prevalence of ESBL production in this species which was 2.9% in the Italian study [32], 6% in the Chinese study [33], and 12.8% in the Korean study [8]. This observation could only be explained on the fact that all these *Enterobacter cloacae* isolates were recovered from King Abdullah University Teaching Hospital during a period of outbreak, as supported by their semi-identical antibiogram profiles and DDD test results.

In *Escherichia coli* isolates, ESBL production was found in 21 out of a total of 195 isolates recovered (10.8%). This percentage agrees with the Chinese study, which demonstrated ESBL production in 11.4% of their *Escherichia coli* isolates [33], the Saudi Arabian study (9.6%) [34], and the Kuwaiti study (11.7%) [35], but higher than that of the Italian study (1.2%) [32], and lower than the Taiwanese one [11]. It is worth noting that most ESBL-producing *E. coli* isolates were from King Abdullah University Teaching Hospital, whereas those from PBTH and PRTH did not exceed 6%.

In *Klebsiella oxytoca* isolates, ESBL production was found in 8/28 (28.5%) of the isolates recovered. This percentage (although the sample size is not large enough) is considered to be high when compared to those for *Klebsiella oxytoca* worldwide, where the Italian study reported 15% prevalence [32]. It is also worth noting that isolates from KAUTH showed the highest degree of ESBL production (80%, 4/5), whereas PRTH and PBTH showed ESBL production in 2/9 isolates (22.2%), and 2/14 (14.3%), respectively. Furthermore, ESBL started to appear among *Citrobacter frundii, Acinetobacter calcoaceticus, Enterobacter hormaechei,* and *Stenotrophemonas maltophilia* isolates from the tertiary care hospital KAUTH, which may reflect the expansion of the genes coding for ESBLs production to other bacterial genera. Although their prevalence in our samples is not high, however, these organisms are becoming a threat to ICU patients and becoming involved in nosocomial infections as reported in Taiwanese, Bulgarian, and Korean hospitals [8, 9, 11]. 

The NCCLS (CLSI) [27] recommend testing for ESBL production using CTX, CAZ, ATM, CRO, and CPD in combination with AMC. In our study, we have evaluated the effectiveness of each of the above antibiotics in addition to ZOX in detection of ESBL production by the DDD method.

In *Escherichia coli*, both CTX and ZOX detected 20/21 (95%) of the ESBL-producing isolates, whereas ATM detected only 85.7% of the isolates. However, CAZ, CRO, CPD were not efficient in detecting ESBL-producing *E. coli.* Similarly, ZOX, CTX, and ATM were efficient in detecting 95%, 81.7%, 71.7% ESBL-producing *Klebsiella pneumonia*, respectively. The other antibiotics could detect few ESBL-producing *K. pneumoniae.*

In *Klebsiella oxytoca,* ZOX showed excellent performance by detecting 7/8 (87.5) of the ESBL-producing isolates. ATM detected 75% of the isolates, while the other antibiotics detected only 50% or less of the ESBL-production in the isolates.

In *Enterobacter cloacae*, CTX and CRO performed excellently by detecting 14/15 (93.3%) of the ESBL producing isolates, whereas CAZ and ZOX detected 13/15 (86.7%) of the ESBL producers, while CPD detected only 12/15 (80%) of them. 

In conclusion, ESBL-producing gram negative bacteria constituted 22.9% (108/472) of all recovered isolates, and were prevalent among *E. coli, K. pneumoniae, K. oxytoca, Enterobacter cloacae*, especially from samples collected from patients in secondary and tertiary care hospitals. The inclusion of ceftizoxime (ZOX) to the ESBL-detection panel will increase the efficacy of the DDD test in detecting ESBL producers.

## Figures and Tables

**Table 1:** Bacterial species isolated from clinical samples.

Bacterial species	Blood culture	CSF	Sputum	Swab	Urine	Miscellaneous	Total
*Acinetobacter baumannii*						2	2
*Acinetobacter calcoaceticus*	6		1			2	9
*Acinetobacter lwoffii*	1						1
*Acinetobacter species*			1	2	1	2	6
*Alcalgenes xylosoxidans*		1					1
*Citrobacter frundii*	1			1	6		8
*Escherichia coli*	6	1		13	171	4	195
*Enterobacter aerogenes*					1		1
*Enterobacter cloacae*	11		1		5		17
*Enterobacter hormaechei *					1	1	2
*Enterobacter species*				7	4	1	12
*Klebsiella pneumoniae*	1			8	19		28
*Klebsiella oxytoca*	48	1	2	9	21	3	84
*Proteus mirabilis*				6	16	1	23
*Pseudomonas aeruginosa*	4	12	3	24	10	11	64
*Pseudomonas species*		1		10	4	1	16
*Serratia marcescens *					1		1
*Stenotrophemonas maltophilia*					2		2
*Total*	79	16	8	80	262	27	472

Miscellaneous: Aspirate, Bone, Cornea, Peritoneal, Tissues, Tracheostomy.

**Table 2:** ESBL production among the gram-negative bacteria.

	*Acinetobacter calcoaceticus *	*Citrobacter frundii*	*Escherichia coli*	*Enterobacter cloacae*	*Enterobacter hormaechei *	*Klebsiella oxytoca*	*Klebsiella pneumoniae *	*Stenotrophemonas maltophilia*
Total isolate	9	8	195	17	2	28	84	2
Number ESBL producer	1	1	21	15	1	8	60	1
Percentage ESBL producer/species	11.1	12.5	10.8	88.2	50	28.6	71.4	50

**Table 3:** Efficasy of cephalosporins in detecting ESBL-producers.

Species (count)	Interpretation	AMC	CTX	CAZ	ATM	CRO	ZOX	CPD	CTT	FEP	CXM	CFP
*Acinetobacter calcoaceticus* (1)	**S**		1				1		1	1		
	**I**					1						1
	**R**	1		1	1			1			1	
	**A**		**1**	**1**	**1**	**1**	**1**	**1**		**1**	**1**	**1**

*Citrobacter frundii* (1)	**S**								1			
	**I**											
	**R**	1	1	1	1	1	1	1		1	1	1
	**A**		**1**				**1**			**1**		

*Escherichia coli* (21)	**S**	7		6			8		21	4		
	**I**	11	3	4	4	3	9			6	1	1
	**R**	3	18	11	17	18	4	21		11	20	20
	**A**		**20**	**15**	**18**	**11**	**20**	**4**	**7**	**18**	**4**	**11**

*Enterobacter cloacae* (15)	**S**						12		3	12		1
	**I**		12	1		12	2		1	1		11
	**R**	15	3	14	15	3	1	15	11	2	15	3
	**A**		**14**	**13**	**15**	**14**	**13**	**12**	**1**	**15**	**12**	**13**

*Enterobacter hormaechei* (1)	**S**											
	**I**						1					
	**R**	1	1	1	1	1		1		1	1	1
	**A**						**1**					

*Klebsiella oxytoca* (8)	**S**			1			2		7	1		
	**I**	5		1	1		3		1	1		
	**R**	3	8	6	7	8	3	8		6	8	8
	**A**		**4**	**3**	**6**	**2**	**7**			**6**		**4**

*Klebsiella pneumonia* (60)	**S**	8		27	1		31		60	17	1	
	**I**	37	16	9	2	15	29			8	5	9
	**R**	15	44	24	57	49		60		35	54	51
	**A**		**49**	**36**	**43**	**26**	**57**	**15**	**10**	**46**	**11**	**31**

*Stenotrophemonas maltophilia* (1)	**S**	1	1	1					1	1		1
	**I**				1	1						
	**R**						1	1			1	
	**A**				**1**	**1**	**1**	**1**				

AMC: Amoxicillin-Clavulanic Acid, CTX: Cefotaxime, CAZ: Ceftazidime, ATM: Aztreonam, CRO: Ceftriaxone, ZOX: Ceftizoxime, CPD: Cefpodoxime, CTT: Cefotetan, FEP: Cefepime, CXM: Cefuroxime, and CFP: Cefoperazone, S: Sensitive, I: Intermediate, R: Resistant, A: Augmentation.
